# A proposal for a comprehensive approach to infections across the surgical pathway

**DOI:** 10.1186/s13017-020-00295-3

**Published:** 2020-02-18

**Authors:** Massimo Sartelli, Leonardo Pagani, Stefania Iannazzo, Maria Luisa Moro, Pierluigi Viale, Angelo Pan, Luca Ansaloni, Federico Coccolini, Marcello Mario D’Errico, Iris Agreiter, Giorgio Amadio Nespola, Francesco Barchiesi, Valeria Benigni, Raffaella Binazzi, Stefano Cappanera, Alessandro Chiodera, Valentina Cola, Daniela Corsi, Francesco Cortese, Massimo Crapis, Francesco Cristini, Alessandro D’Arpino, Belinda De Simone, Stefano Di Bella, Francesco Di Marzo, Abele Donati, Daniele Elisei, Massimo Fantoni, Anna Ferrari, Domitilla Foghetti, Daniela Francisci, Gianni Gattuso, Andrea Giacometti, Guido Cesare Gesuelli, Cristina Marmorale, Enrica Martini, Marcello Meledandri, Rita Murri, Daniela Padrini, Dalia Palmieri, Paola Pauri, Carla Rebagliati, Enrico Ricchizzi, Vittorio Sambri, Anna Maria Schimizzi, Walter Siquini, Loredana Scoccia, Giancarlo Scoppettuolo, Gabriele Sganga, Nadia Storti, Marcello Tavio, Giulio Toccafondi, Fabio Tumietto, Bruno Viaggi, Marco Vivarelli, Cristian Tranà, Melina Raso, Francesco Maria Labricciosa, Sameer Dhingra, Fausto Catena

**Affiliations:** 1 Department of Surgery, Macerata Hospital, ASUR Marche, Macerata, Italy; 2 Infectious Diseases Unit, Bolzano Central Hospital, Bolzano, Italy; 3 Ministry of Health, Rome, Italy; 4 Regional Agency for Health and Social Care, Emilia-Romagna Region–ASSR, Bologna, Italy; 5 Department of Medical and Surgical Sciences, Clinics of Infectious Diseases, S. Orsola-Malpighi Hospital, “Alma Mater Studiorum”-University of Bologna, Bologna, Italy; 6 Infectious Diseases, ASST di Cremona, Cremona, Italy; 7 General, Emergency and Trauma Surgery Department, Bufalini Hospital, Cesena, Italy; 8 Emergency Surgery Unit, New Santa Chiara Hospital, University of Pisa, Pisa, Italy; 9 Department of Biomedical Sciences and Public Health, Marche Polytechnic University, Ancona, Italy; 10 Bone Marrow Transplant Unit, Denis Burkitt, St. James’s Hospital, Dublin, Ireland; 11 Infectious Diseases Unit, Fermo Hospital, ASUR Marche, Marche, Italy; 12 Infectious Diseases Unit, Azienda Ospedaliera Ospedali Riuniti Marche Nord, Pesaro, Italy; 13 Clinical Administration, Senigallia Hospital, ASUR Marche, Senigallia, AN Italy; 14 Infectious Diseases Clinic, Department of Medicine, “S. Maria” Hospital, Terni, University of Perugia, Perugia, Italy; 15 Infectious Diseases Unit, Macerata Hospital, ASUR Marche, Macerata, Italy; 16 Department of Hospital Pharmacy, Ospedali Riuniti di Ancona, Ancona, Italy; 17 Department of Anesthesiology and Intensive Care Unit, Civitanova Marche Hospital, ASUR Marche, Civitanova Marche, MC Italy; 18 Emergency Surgery and Trauma Care Unit, San Filippo Neri Hospital, Rome, Italy; 19 Infectious Diseases Unit, Pordenone Hospital, Pordenone, Friuli-Venezia Giulia Italy; 20 Infectious Diseases Unit, Rimini Hospital, Rimini, Italy; 21 Hospital Pharmacy Unit, Santa Maria della Misericordia Hospital, Azienda Ospedaliera di Perugia, Perugia, Italy; 22 Operative Unit of General Surgery, Azienda USL IRCCS Reggio Emilia, Reggio Emilia, Italy; 23 Infectious Diseases Department, Trieste University Hospital, Trieste, Italy; 24 Department of Surgery, Versilia Hospital, Lido di Camaiore, Italy; 25 Department of Anesthesiology and Intensive Care Unit, Department of Biomedical Sciences and Public Health, Università Politecnica delle Marche, Ancona, Italy; 26 Department of Anesthesiology and Intensive Care Unit, Macerata Hospital, ASUR Marche, Macerata, Italy; 27 Department of Infectious Diseases, Fondazione Policlinico A. Gemelli IRCCS, Istituto di Clinica delle Malattie Infettive, Università Cattolica S. Cuore, Rome, Italy; 28 Department of Critical Care Medicine Unit, San Filippo Neri Hospital, Rome, Italy; 29 Department of Surgery, Azienda Ospedaliera Ospedali Riuniti Marche Nord, Pesaro, Italy; 30 Infectious Diseases Clinic, University of Perugia, Perugia, Italy; 31 Infectious Diseases Unit, Carlo Poma Hospital, Mantua, Italy; 32 Infectious Diseases Clinic, Department of Biological Sciences and Public Health, Marche Polytechnic University, Ancona, Italy; 33 Department of Surgery, Marche Polytechnic University of Marche Region, Ancona, Italy; 34 Hospital Hygiene Unit, Azienda Ospedaliero-Universitaria Ospedali Riuniti, Ancona, Italy; 35 Unit of Microbiology and Virology, San Filippo Neri Hospital, Rome, Italy; 36 Clinical Administration Santa Maria Annunziata Hospital, USL Toscana Centro, Florence, Italy; 37 Epidemiology Office, AUSL Pescara, Pescara, Italy; 38 Unit of Microbiology and Virology, Senigallia Hospital, Senigallia, AN Italy; 39 Surgical Unit, Savona Hospital, Savona, Italy; 40 Department of Experimental, Diagnostic and Specialty Medicine (DIMES), University of Bologna, Bologna, Italy; 41 Unit of Microbiology, The Great Romagna Area Hub Laboratory, Pievesestina, Cesena, Italy; 42 Department of Internal Medicine, Jesi Hospital, Ancona, Italy; 43 Unit of Hospital Pharmacy, Macerata Hospital, ASUR Marche, Macerata, Italy; 44 Infectious Diseases Unit, Fondazione Policlinico Universitario A. Gemelli IRCCS, Rome, Italy; 45 Division of Emergency Surgery, Fondazione Policlinico Universitario A. Gemelli IRCCS, Università Cattolica del Sacro Cuore, Rome, Italy; 46 General Directory, ASUR Marche, Ancona, Italy; 47 Infectious Diseases Unit, Azienda Ospedaliero Universitaria Ospedali Riuniti, Ancona, Italy; 48 Clinical Risk Management and Patient Safety Center, Tuscany Region, Florence, Italy; 49 Department of Anesthesiology, Neuro Intensive Care Unit, Florence Careggi University Hospital, Florence, Italy; 50 Unit of Hepato-Pancreato-Biliary and Transplant Surgery, Department of Experimental and Clinical Medicine, Polytechnic University of Marche, Ancona, Italy; 51 Health First Europe, Brussels, Belgium; 52 Global Alliance for Infections in Surgery, Porto, Portugal; 53 Faculty of Medical Sciences, School of Pharmacy, The University of the West Indies, St. Augustine, Trinidad and Tobago; 54 Emergency Surgery Department, Parma University Hospital, Parma, Italy

**Keywords:** Antimicrobial resistance, Antimicrobial stewardship, Healthcare-associated infections, Infection control, Multidrug-resistant organisms, Preoperative antibiotic prophylaxis, Surgical site infections

## Abstract

Despite evidence supporting the effectiveness of best practices in infection prevention and management, many healthcare workers fail to implement them and evidence-based practices tend to be underused in routine practice. Prevention and management of infections across the surgical pathway should always focus on collaboration among all healthcare workers sharing knowledge of best practices. To clarify key issues in the prevention and management of infections across the surgical pathway, a multidisciplinary task force of experts convened in Ancona, Italy, on May 31, 2019, for a national meeting. This document represents the executive summary of the final statements approved by the expert panel.

## Background

Prevention and management of infections across the surgical pathway should always focus on collaboration among all healthcare professionals with shared knowledge and widespread diffusion of best practices.

Leading international organizations, such as the World Health Organization (WHO), acknowledge that collaborative practice is essential for achieving a concerted approach to providing care that is appropriate to meet the needs of patients, thus optimizing individual health outcomes and overall service delivery of healthcare [1].

## Methods

To clarify key issues in the prevention and management of infections across the surgical pathway, a multidisciplinary task force of national experts convened in Ancona, Italy, on May 31, 2019, for a national meeting. The multifaceted nature of these infections has led to a multidisciplinary collaboration involving epidemiologists and infection control specialists, infectious disease specialists, hospital pharmacists, microbiologists, intensivists, general and emergency surgeons, and nurses. During the meeting, the panelists presented the statements developed for each of the main questions regarding the prevention and management of infections in surgery. An agreement on the statements was reached by the Delphi method. Statements were approved with an agreement of ≥ 80%. After the meeting, the expert panel met via email to prepare and revise the consensus paper resulting from the meeting. The manuscript was successively reviewed by all members and ultimately revised as the present manuscript. This document represents the executive summary of the final statements approved by the expert panel.

## Healthcare-associated infections and patient safety

Improving patient safety in hospitals worldwide presently requires a systematic approach to preventing healthcare-associated infections (HAIs) and antimicrobial resistance (AMR). The two go together. HAIs are infections that occur while receiving healthcare. Patients with medical devices (central lines, urinary catheters, ventilators) or who undergo surgical procedures are at risk of acquiring HAIs.

The occurrence of HAIs continues to escalate at an alarming rate. These infections result in significant patient illnesses and deaths, prolong the duration of hospital stay, and necessitate additional diagnostic and therapeutic interventions, which generate supplementary costs to those already sustained due to the patient’s underlying disease. However, the phenomenon is not yet sufficiently perceived among both healthcare workers (HCWs) and patients, thus resulting in a low level of intervention request and relative inadequate responses [2]. Although HAIs are the most frequent adverse events in healthcare, their true global burden remains unknown because of the difficulty in gathering reliable data: most countries lack surveillance systems for HAIs, and those do have them struggle with the complexity and the lack of uniformity of criteria [3].

HAIs are considered adverse events, and as many are preventable, they are considered an indicator of the quality of patient care and a patient safety issue. In 2018, a systematic review and meta-analysis of studies between 2005 and 2016 evaluated the results of multifaceted interventions to reduce catheter-associated urinary tract infections (CAUTIs), central line-associated bloodstream infections (CLABSIs), surgical site infections (SSIs), ventilator-associated pneumonia, and hospital-acquired pneumonia not associated with mechanical ventilation in acute care or long-term care settings [4]. Of the 5226 articles identified, 144 studies were included in the final analysis. Published evidence suggested a sustained potential for the significant reduction of HAI rates in the range of 35–55% associated with multifaceted interventions irrespective of a country’s income level.

**Question 1. How can you implement global guidelines for the prevention of surgical site infections (SSIs)?**

**Statement 1.1. Recent global guidelines for the prevention of SSIs can support healthcare workers to develop or strengthen infection prevention and control programs, with a focus on surgical safety, as well as AMR action plans. All healthcare workers should adopt these evidence-based recommendations in their clinical practice.**

**Statement 1.2. A safer surgical care requires a range of precautions aimed at reducing the risk of SSIs before, during and after surgery.**

**Statement 1.3. To support local implementation of guidelines for the prevention of SSIs, 5 steps of a multimodal strategy, including system change, training and education, evaluation and feedback, communications for awareness raising and institutional safety climate and culture are suggested.**

Improving behavior in infection prevention and control (IPC) practices remains a challenge. Despite progress in preventive knowledge, SSIs remain the most common HAI among surgical patients and one of the most frequent adverse events in hospitals. They represent a major clinical problem in terms of morbidity, mortality, length of hospital stay, and overall direct and not direct costs worldwide. It is obviously important to improve patient safety by reducing the occurrence of SSIs. Preventing SSIs is a global priority, also because bacteria are becoming increasingly resistant to antibiotics, making SSI prevention even more important nowadays. On the other hand, SSI prevention is complex and requires the integration of a range of measures before, during, and after surgery.

Both WHO [5, 6] and the Centers for Disease Control and Prevention (CDC) [7] have published guidelines for the prevention of SSIs. The 2016 WHO Global guidelines for the prevention of SSI [5, 6] are evidence-based including systematic reviews presenting additional information in support of actions to improve practice. The first-ever global guidelines for the prevention of SSIs were published on November 3, 2016, then were updated in some parts and published in a new edition in December 2018. The guidelines include 13 recommendations for the preoperative period and 16 for preventing infections during and after surgery. They range from simple precautions such as ensuring that patients bathe or shower before surgery, appropriate way for surgical teams to clean their hands, guidance on when to use prophylactic antibiotics, which disinfectants to use before incision, and which sutures to use.

The proposed recommendations are classified as follows:

- “Strong”: Expert panel was confident that benefits outweighed risks, considered to be adaptable for implementation in most (if not all) situations, and patients should receive intervention as a course of action.
- “Conditional”: Expert panel considered that benefits of intervention probably outweighed the risks; a more structured decision-making process should be undertaken, based on stakeholder consultation and involvement of patients and healthcare professionals.

In 2018, WHO published a document about the implementation approaches for these evidence-based recommendations [8]. The purpose of this document is to present a range of tested approaches to achieve successful SSI prevention implementation at the facility level, including in the context of a broader surgical safety climate, using an evidence- and team-based approach and a multimodal strategy for achieving sustainable change based on system change, training and education, evaluation and feedback, communications for awareness raising, and institutional safety climate and culture. The manual is aimed at all those concerned with the prevention of SSIs. A multidisciplinary team is necessary to successfully implement preventive measures. This should include at least IPC and associated staff, such as those working in epidemiology, decontamination/sterilization, quality improvement and patient safety, hospital administration, and the surgical teams (including surgeons, anesthesiologists, and perioperative nurses).

**Question 2. Why do you have to survey HAIs?**

**Statement 2.1. Surveillance of HAIs improves the quality of care because it reduces the risk of infection. It should be supported by all healthcare workers.**

IPC program should be in place to prevent HAIs in all hospitals worldwide, and one of the main cornerstones is the presence of a formal system to monitor IPC and ensure that appropriate actions are taken to minimize infection rates [9]. HAI surveillance is a challenging task also because it requires particular expertise after obtaining epidemiological data to assess the quality of the information produced and to interpret its meaning and root cause in order to tailor intervention and prevention measures.

Program surveying SSIs have been implemented throughout the world and are associated with a reduction in SSI rates. Data on non-prosthetic surgery from the Italian SSI surveillance program for the period 2009 to 2011 [10] demonstrated that implementation of a national surveillance program was helpful in reducing SSI rates and should be prioritized in all healthcare systems. A 17% decrease in SSI related to ten selected procedures was reported between 2008 and 2013 in the USA following improvement programs [11]. In African hospitals, a 60% SSI risk reduction was observed following the implementation of a WHO multimodal strategy in the context of the WHO Surgical Unit-based Safety Program (SUSP) including SSI surveillance [12]. Surveillance also allows hospitals and clinicians to measure the effectiveness of strategies that are implemented to decrease infection rates. Infection rate data should be used in a positive way to improve the quality and safety of healthcare.

HAI surveillance is conventionally conducted by two methods. Passive surveillance (self-reporting of suspected HAIs by the treating physicians) is a very poor and inefficient method to track HAIs as there is a risk of bias and underreporting. Active surveillance, on the other hand, is the systematic collection of data by a designated unbiased surveillance team. This is the method recommended by the main surveillance networks. Following the data extraction, analysis of the collected information should be done. Feedback and reports after the analysis should be disseminated by infection control committees, keeping the confidentiality of individuals. The importance of surveillance systems for HAI control has been accepted globally, and some countries have established national surveillance systems with the aim to prevent HAIs.

**Question 3. How can you implement the prevention of HAIs?**

**Statement 3.1. It is necessary to set up a solid and branched surveillance network gathering alert signals, verifying their severity and initiating the organizational response via “warnings”.**

**Statement 3.2. The collection and analysis of monitoring data serve to identify vulnerabilities in the system. This is the basis for organizational improvement, risk reduction, and damage control.**

HAIs affect around 5–15% of all hospital patients worldwide. Despite the availability of standard procedures and evidence-based guidelines aiming at reducing the impact of HAIs, the implementation of those into routine practice appears as the biggest challenge [13].

HAI surveillance and timely feedback of results are strongly recommended by WHO as part of the core components of effective IPC programs [14]. Every healthcare facility should be committed to provide quality and safe care. Surveillance is not to be undertaken in isolation, but as integrated into a comprehensive and multimodal IPC strategy. Conducting high-quality IPC and surveillance is crucial to assess the safety level of the surgical workflow, detect criticalities, and diffuse warnings to trigger the response capability of healthcare organizations. Feedback on IPC achievements should be constantly monitored and timely disseminated throughout the levels of the organization by the hospital IPC [15]. Surveillance of HAIs is a fundamental aspect of the IPC program, in particular, when SSIs are identified as a target for improvement.

Particularly in surgical care, SSI surveillance provides feedback to surgical teams on the HAI risks patients are exposed to. Cooperation of surgical teams in surveillance efforts is crucial to make visible to them the effect on patients’ care, if they have confidence in the methods being used. Thus, it is important for surgeons to comprehend the opportunities of the surveillance process for surgical care improvements [15]. In this regard, the support of human factors and ergonomics paired with implementation science is crucial to embed the knowledge gained through an epidemiological into the daily routine of HCWs [16].

**Question 4. How can you prevent and manage** ***Clostridioides difficile*** **infection (CDI)?**

**Statement 4.1. Key points for** **CDI prevention are:**

- **Antimicrobial stewardship.**
- **Contact precautions.**
- **Hand washing (soap, not alcohol).**
- **Avoid unnecessary gastric acid suppressants.**

**Statement 4.2. Key points for CDI treatment are:**

- **Stop unnecessary antibiotics.**
- **Metronidazole (mild episodes).**
- **Oral/intracolonic vancomycin.**
- **Oral fidaxomicin.**
- **IV bezlotoxumab (recurrent episodes).**
- **Fecal microbiota transplantation.**
- **Prompt surgery when indicated.**

In the last two decades, CDI has become a major global public health problem, with a dramatic increase in the incidence and severity of episodes. CDI may be a particular concern in surgical patients, as surgery may predispose patients to CDI and surgery itself could be necessary to treat severe cases of CDI [17].

Risk factors for CDI may be divided into three general categories [17]:

1. Host factors (immune status, co-morbidities)
2. Exposure to *C. difficile* spores (hospitalizations, community sources, long-term care facilities)
3. Factors that disrupt normal colonic microbiome (antibiotics, other medications, surgery)

The main risk factors are antibiotic exposure, age more than 65 years, comorbidity or underlying conditions, inflammatory bowel diseases, immunodeficiency (including human immunodeficiency virus infection), malnutrition, and low serum albumin level. Antibiotics play a central role in the pathogenesis of CDI, presumably by disrupting the normal gut flora, thereby providing a perfect setting for *C. difficile* to proliferate and produce toxins. Although nearly all antibiotics have been associated with CDI, clindamycin, third-generation cephalosporins, penicillins, and fluoroquinolones have usually been considered at greatest risk [16].

A prompt and precise diagnosis is an important aspect of effective management of CDI. Early identification of CDI allows the establishment of an early treatment and can improve outcomes. Rapid isolation of infected patients is fundamental to limit *C. difficile* transmission. This is particularly important in reducing environmental contamination as spores can survive for months in the environment, despite regular use of environmental cleaning agents. Patients with CDI should be maintained in contact (enteric) precautions until the resolution of diarrhea (passage of formed stool for at least 48 h). Patients with known or suspected CDI should ideally be placed in a private room with en suite hand washing and toilet facilities. If a private room is not available, as often occurs, known CDI patients may be cohort, nursed in the same area, though the theoretical risk of transfection with different strains exists. Hand hygiene with soap and water and the use of contact precautions along with a good cleaning and disinfection of the environment and patient equipment should be used by all HCWs contacting any patient with known or suspected CDI. Alcohol-based hand sanitizers are highly effective against non-spore-forming organisms, but they may not kill *C. difficile* spores or remove *C. difficile* from the hands. The most effective way to remove them from the hands is through handwashing with soap and water.

In cases of suspected severe CDI, antibiotic agents should be discontinued, if possible [18]. A meta-analysis addressing factors associated with prolonged symptoms and severe disease due to *C. difficile* showed that continued use of antibiotics for infections other than CDI is significantly associated with an increased risk of CDI recurrence [18]. If continued antibiotic therapy is required for the treatment of the primary infection, antimicrobial therapy with agents that are less frequently implicated with antibiotic-associated CDI should be used; these include parenteral aminoglycosides, sulfonamides, macrolides, vancomycin, or tetracycline/tigecycline.

Although there is a clinical association between proton pump inhibitor (PPI) use and CDI [19], no randomized controlled trial (RCT) studies have studied the relationship between discontinuing or avoiding PPI use and the risk of CDI. Thus, a strong recommendation to discontinue PPIs in patients at high risk for CDI regardless of the need for PPIs will require further evidence. However, stewardship activities to discontinue unneeded PPIs are strongly warranted.

Regarding treatment, antibiotic therapy is the first choice for CDI treatment and molecule choice should be based according to the severity of the disease. When antibiotic therapy is indicated for symptomatic cases with a positive stool *C. difficile* test, options include metronidazole, oral or intraluminal vancomycin, and oral fidaxomicin [20–24]. Metronidazole should be limited to the treatment of an initial episode of mild-moderate CDI. Vancomycin orally 125 mg four times daily for 10 days is considered superior to metronidazole in severe CDI [25–27]. Doses of up to 500 mg have been used in patients with severe or fulminant, as defined as hypotension or shock, ileus, or megacolon, CDI [28], although there is little evidence for this in the literature. Fidaxomicin orally 200 mg twice daily for 10 days may be a valid alternative to vancomycin in patients with CDI [29, 30]. Fidaxomicin may be useful for treating patients who are considered at high risk for recurrence (elderly patients with multiple comorbidities who are receiving concomitant antibiotics).

Fecal microbiota transplantation (FMT) is an effective option for patients with multiple CDI recurrences who have failed appropriate antibiotic treatments [31]. FMT involves infusing intestinal microorganisms (in a suspension of healthy donor stool) into the intestine of patients to restore the intestinal microbiota. The rationale of FMT is that disruption of the normal balance of colonic flora allows *C. difficile* strains to grow and produce CDI. By reintroducing normal flora via donor feces, the imbalance may be corrected and normal bowel function re-established [31].

Coadjuvant treatment with monoclonal antibodies (bezlotoxumab) may prevent recurrences of CDI, particularly in patients with CDI due to the 027 epidemic strain, in immunocompromised patients and in patients with severe CDI. Bezlotoxumab (MK-6072), approved in 2016 by Food and Drug Administration (FDA), is a human monoclonal antibody which reduces recurrent CDI by blocking the binding of *C. difficile* toxin B to host cells, thus limiting epithelial damage and facilitating recovery of the microbiome [32].

Patients with severe CDI who progress to systemic toxicity should undergo early surgical consultation and should be evaluated for potential surgical intervention. Resection of the entire colon should be considered to treat patients with fulminant colitis. However, diverting loop ileostomy with colonic lavage is a useful alternative to resection of the entire colon.

**Question 5. How can you prevent central-venous catheter-related infections?**

**Statement 5.1. The most effective means to reduce to the minimum possible central-venous catheter-related infections are represented by a «bundles» management, based on the guidelines, implemented with training and motivational meetings aimed at increasing compliance of healthcare workers (better if organized in a dedicated team) and applied by checklist.**

In order to guarantee a correct management of central venous catheter-related infections, a correct diagnostic framework is essential, to be obtained by a standardized execution of blood cultures from a peripheral vein and central venous catheter (CVC), in order to be able to implement a correct interpretation of the results and take timely decisions on a possible removal/conservative strategy towards the catheter.

About half of nosocomial bloodstream infections occur in intensive care units (ICUs), and the majority of them are associated with an intravascular device. Central venous catheter-related bloodstream infections (CRBSIs) are an important cause of HAIs. CVCs are integral to modern clinical practices and are inserted in critically ill patients for the administration of fluids, blood products, medication, and nutritional solutions and for hemodynamic monitoring. They are the main source of bacteremia in hospitalized patients and therefore should be used only if necessary.

Risk factors for CRBSIs include patient-, catheter-, and operator-related factors. Several factors have been proposed to participate in the pathogenesis of CRBSI. Hospitalized patients with neutropenia are at higher risk. However, other host risk factors also include immune deficiencies in general, chronic illness, and malnutrition. The diagnosis of CRBSI is often suspected clinically in a patient using a CVC who presents with fever or chills, unexplained hypotension, and no other localizing sign. Diagnosis of CRBSI requires establishing the presence of bloodstream infection and demonstrating that the infection is related to the catheter. However, blood cultures should not be drawn solely from the catheter port as these are frequently colonized with skin contaminants, thereby increasing the likelihood of a false-positive blood culture. Indeed, according to IDSA guidelines [33], a definitive diagnosis of CRBSI requires a culture of the same organism from both the catheter tip and at least one percutaneous blood culture. Alternatively, the culture of the same organism from at least two blood samples (one from a catheter hub and the other from a peripheral vein or second lumen) meeting criteria for quantitative blood cultures or differential time to positivity. Most laboratories do not perform quantitative blood cultures, but many laboratories are able to determine the differential time to positivity. Quantitative blood cultures demonstrating a colony count from the catheter hub sample ≥ 3-fold higher than the colony count from the peripheral vein sample (or a second lumen) supports a diagnosis of CRBSI. Differential time to positivity refers to growth detected from the catheter hub sample at least 2 h before growth detected from the peripheral vein sample. The CVC and arterial catheter, if present, should be cultured and removed as soon as possible if the patient has unexplained sepsis or erythema overlying the catheter insertion site or purulence at the catheter insertion site in immunocompromised patients.

Antibiotic therapy for catheter-related infection is often initiated empirically. The initial choice of antibiotics will depend on the severity of the patient’s clinical disease, the risk factors for infection, and the likely pathogens associated with the specific intravascular device. Resistance to antibiotic therapy due to biofilm formation also has an important role in the management of bacteremia. In fact, the nature of the biofilm structure makes microorganisms difficult to eradicate and confer an inherent resistance to antibiotics.

CRBSIs can be reduced by a range of interventions including closed infusion systems, aseptic technique during insertion and management of the central venous line, early removal of central venous lines, and appropriate site selection. Different measures have been implemented to reduce the risk for CRBSI, including the use of maximal barrier, precautions during catheter insertion, effective cutaneous anti-sepsis, and preventive strategies based on inhibiting microorganisms originating from the skin or catheter hub from adhering to the catheter [34]. The simultaneous application of multiple recommended best practices to manage CVCs has been associated with significant declines in the rates of CRBSI. Bundles can be defined as the systematic implementation of a set of evidence-based practices, usually three to five, that when performed properly and collectively can improve patient outcomes. Research on CRBSI prevention demonstrated the effectiveness of bundles, which reduce the incidence of CRBSI by up to 80% [35–37], reaching a rate of 0 in some cases [38]. Education and training of healthcare workers and adherence to standardized protocols for insertion and maintenance of intravascular catheters significantly reduced the incidence of catheter-related infections and represent the most important preventive measures.

## The global burden of antimicrobial resistance

AMR has emerged as one of the principal public health problems of the twenty-first century. This has resulted in a public health crisis of international concern. Combating resistance has become a top priority for global policymakers and public health authorities. New mechanisms of resistance continue to emerge and spread globally, challenging our ability to manage common infections. Antibacterial and antifungal use in animal and agricultural industries aggravates selective pressure on microbes. A One Health approach is required urgently. Addressing the rising threat of AMR requires a holistic and multisectoral approach—referred to as One Health—because antimicrobials used to treat various infectious diseases in animals may be the same or similar to those used for humans. Resistant bacteria arising in humans, animals, or the environment may spread from one to another and from one country to another. AMR does not recognize geographic or human-animal borders [39].

The worldwide impact of AMR is significant, in terms of economic and patient outcomes, because of untreatable infections or those necessitating antibiotic agents of last resort leading to increased length of hospital stay, morbidity, death, and treatment cost. Raising awareness of AMR and promoting behavioral change through public communication programs that target different audiences in human health, animal health, and agricultural practice, as well as consumers, are critical to tackling this issue.

HCWs play a central role in preventing the emergence and spread of resistance. An effective and cost-effective strategy to reduce AMR should involve a multifaceted approach aimed at optimizing antibiotic use, strengthening surveillance and IPC, and improving patient and clinician education regarding the appropriate use of antibiotic agents.

Although the phenomenon of AMR can be attributed to many factors, there is a well-established relationship between antimicrobial prescribing practices and the emergence of antimicrobial-resistant pathogens. However, after they have emerged, resistant pathogens may be transmitted from one individual to another. Every infection prevented is one that needs no treatment. Prevention of infection can be cost-effective and implemented in all settings and sectors, even where resources are limited. A range of factors such as diagnostic uncertainty, fear of clinical failure, time pressure, or organizational contexts can complicate both antibiotic prescribing decisions and preventing measures. Because of cognitive dissonance (recognizing that action is necessary but not implementing it), however, changing behavior is extremely challenging, and awareness of AMR is still low.

Every hospital worldwide should utilize the existing resources to create an effective multidisciplinary team for combating AMR. The best strategies for combating AMR are not definitively established and are likely to vary based on local culture, policy, and routine clinical practice despite several guidelines on the topic.

## The Italian situation

In a study published in January 2019 in The Lancet Infectious Diseases, the European Center for Disease Prevention and Control (ECDC) assessed the weight of infections due to multiresistant bacteria in invasive isolates in Europe [40].

Elaborating the 2015 data contained in the European Antimicrobial Resistance Surveillance Network (EARS-Net) and crossing them with a conversion factor, the authors arrived at the first estimate of the impact of antibiotic resistance on the European population. The authors estimate that infections caused by multiresistant bacteria can cause at least 33,000 deaths each year in Europe (equal to the sum of deaths caused by influenza, AIDS, and tuberculosis) and almost 880.000 cases of disability. Italy and Greece have the most infections from multiresistant bacteria. Although we consider that the Italian population is of a medium-high age, it is noteworthy that about a third of deaths due to antibiotic-resistant bacterial infections in Europe have been in Italy. Not surprisingly, in December 2017, the ECDC published a report on the Italian situation and activities for the prevention and control of antibiotic resistance [41]. The report summarizes visits and meetings that ECDC experts had in Italy from January 9 to 13, 2017, to discuss and specifically assess the situation in the country regarding antibiotic-resistance prevention and control in our country. Observations of this visit by the ECDC confirm that the antibiotic resistance situation in Italian hospitals will represent a serious threat to public health for the country in the near future.

ECDC experts noted the following:

- Little sense of urgency about the current AMR situation from most stakeholders and a tendency by many stakeholders to avoid taking charge of the problem
- Lack of institutional support at the national, regional, and local level
- Lack of professional leadership at each level
- Lack of accountability at each level
- Lack of coordination of the activities between and within levels

According to a report by the Organization for Economic Co-operation and Development (OECD) [42], in Italy, the proportion of antibiotic-resistant infections have grown from 17% in 2005 to 30% in 2015 and will reach 32% in 2030, if antibiotic consumption will continue to follow the same trends. The proportion of antibiotic resistance in Italy is substantially higher than that in the 17% average resistance of OECD countries in 2015.

On November 2, 2017, the Ministry of Health published the national anti-microbial resistance plan (PNCAR) 2017–2020 [43], which identified strategies and actions to be implemented at different levels: national, regional, and local. The PNCAR is developed according to a One Health approach. The actions set out in the plan, at the level of central, regional, and local institutions, pursue specific objectives:

- Improve awareness and education of health professionals, citizens, and stakeholders
- Monitor the phenomenon of antibiotic resistance and the use of antibiotics
- Improve IPC
- Optimize the use of antimicrobials in the field of human and animal health
- Increase and support research and innovation.

## Appropriate management of infections across the surgical pathway

Antibiotics can be life-saving when treating bacterial infections but are often used inappropriately. Although most clinicians are aware of the problem of AMR, most underestimate this problem in their own hospital. Clinicians should always optimize antimicrobial management to maximize the clinical outcome of the patients and minimize the emergence of AMR. The necessity of formalized systematic approaches to the optimization of antibiotic therapy in the setting of surgical units worldwide, both for elective and emergency admissions, has become increasingly urgent.

Below, we report 11 strategies for a correct antibiotic therapy.

1. Communication and education.
2. Updating local epidemiological data stratifying them for specific settings.
3. Start and choice treatment always using a severity driven approach.
4. Drafting local algorithms / bundles.
5. Avoid redundant prescriptions.
6. Not being impulsive in starting antimicrobial therapy.
7. Being parsimonious with combination regimens.
8. Strict collaboration with microbiology laboratory in daily life.
9. Being aware about PK/PD issues.
10. Shortening therapy.
11. Creating a multidisciplinary team for specific setting, syndromes, etc.

Hospital-based programs dedicated to improving antibiotic use, commonly referred to as Antimicrobial stewardship programs (ASPs), can both optimize the treatment of infections and minimize adverse events associated with antibiotic use and AMR [44, 45]. Every hospital worldwide should utilize the existing resources to create an effective multidisciplinary team. The preferred means of improving antibiotic stewardship should involve a comprehensive program that incorporates collaboration between various specialties within a healthcare institution including infectious disease specialists, hospital pharmacists, clinical pharmacologists, administrators, epidemiologists, IPC specialists, microbiologists, surgeons, anesthesiologists, intensivists, and underutilized but pivotal stewardship team members, the surgical, anesthetic, and intensive care nurses in our hospitals.

Antimicrobial stewardship policies should be based on both international and national antibiotic guidelines and tailored to local microbiology and resistance patterns. Facility-specific treatment recommendations, based on the guidelines and local formulary options promoted by the antimicrobial stewardship team, can guide clinicians in antibiotic selection and duration of the therapy for the most common indications. Standardizing a shared protocol of antibiotic prophylaxis should represent the first step of any ASP. Since physicians are primarily responsible for the decision to use antibiotics, educating them and changing the attitudes and knowledge that underlie their prescribing behavior are crucial for improving antibiotic prescription. Education is fundamental to every ASP; however, due to cognitive dissonance (recognizing that action is necessary but not implementing it), changing the prescribing behavior is extremely challenging [46]. Efforts to improve educational programs are thus required, and this should preferably be complemented by active interventions such as prospective audits and feedback to clinicians to stimulate further change [47]. It is also crucial to incorporate fundamental ASP and IPC principles in under- and post-graduate training at medical faculties to equip young doctors and other healthcare professionals with the required confidence, skills, and expertise in the field of antibiotic management.

**Question 6. The clinical microbiology laboratory: which is its role in the control of infections with multidrug-resistant bacteria?**

**Statement 6.1. The implementation of microbiological diagnostic activities improves the diagnostic capacity towards infections caused by multidrug-resistant organisms (MDROs).**

**Statement 6.2. The three main challenges of a modern microbiology laboratory are: to maintain high-quality services, to consolidate laboratory medical care into large hospital systems and, as a consequence of consolidation to reach a full automation possibly in all the analytical steps of the diagnosis.**

ASPs and prescribing physicians depend on the information and guidance from the clinical microbiology laboratory, thus making the laboratory vital to patient care and the success of ASPs. The three most relevant challenges of a modern microbiology laboratory are the following.

### Maintaining high-quality and cost-effective services

ASPs aid physicians in providing optimal antimicrobial therapy to their patients, prescribing the right antimicrobial regimen to the right patient for the right period of time, and avoiding the unnecessary use of antimicrobial. Ultimately, ASPs aim to improve patient outcomes while limiting adverse drug events and reducing AMR. The clinical microbiology laboratory plays a critical role in the success of the antimicrobial stewardship efforts by providing essential information for accurately diagnosing and treating patients with infectious diseases [48].

Clinical microbiology laboratories (CMLs) conduct surveillance on the local AMR trends among microbial pathogens. The collection, organization, and communication of resistance data culminates could be summarized in the preparation of the antibiogram. Antibiograms provide critical information to ASPs and to prescribing physicians on local institution susceptibility patterns. CMLs provide patient-specific information by identifying the microbial pathogens and performing the antimicrobial susceptibility testing. This information is necessary so that empiric antimicrobial therapy can be shortened and substituted by a pathogen-driven approach. Over the past decade, there have been several advances in rapid microbiological diagnostic testing. Compared to standard techniques that require 48–72 h for final results, these methods can greatly reduce the time to pathogen identification by providing final organism identification within hours from the sample collection or, less efficiently, from the availability of an isolated bacterial colony by culture-based methods.

### Consolidation of laboratory medical care into large hospital systems: consolidated hospital network systems open the possibility to merger microbiology diagnostic activities into larger laboratories

Many new innovative microbiological diagnostic approaches have been made available during the last 10 years with a major impact on patient care and public health surveillance [49]. In parallel, to enhance the cost-effectiveness of the CMLs, European laboratory professionals have streamlined their organization leading to the amalgamation of diagnostic activities and thus restructuring of their professional relationships with clinicians and public health specialists. Through this consolidation process, an operational model has emerged that combines large centralized CMLs performing a large panel and number of tests within a high-throughput analytical platform connected to several distal laboratories dealing locally with urgent analyses at the near point of care testing. The centralization of diagnostic services so that encompassing a large geographical region has given rise to the concept of regional-scale “microbiology laboratories network” or, in another word to “geographically spread laboratories.” Although the volume-driven cost savings associated with such laboratory networks seem self-evident, the consequences for the quality of patient care and infectious disease surveillance and control remain a challenge even if the fast-changing landscape of CMLs in Europe may give a range of opportunities to contribute to improving the quality of patient care but also the early detection and enhanced surveillance of public health threats caused by infectious diseases.

### Full automation is currently being required to meet the needs of a changing healthcare system based on consolidated geographically spread microbiology laboratories

During the last decade, most CMLs have encountered many management and financial difficulties, mainly resulting from the gradual and continuous increase in sample volume with limited budgets and personnel shortages. Thus, laboratories have been forced to optimize their workflow to raise their productivity: this improvement must be accompanied by at least a maintained analytical quality, but possibly by an improved clinical value of the generated data. Automation was introduced many years ago in several diagnostic disciplines such as chemistry, clinical pathology, and hematology to increase laboratory productivity and quality. The automation process was by far more complicated in molecular biology and bacteriology settings: this was due to several reasons, including the complexity and variability of sample types, the many different analytical processes, and the insufficient volume of samples. However, the introduction of automation was considered to be also applicable in microbiology in more recent years thanks to the technological improvements currently available. Recently, these new technologies have triggered the development of automated solutions specifically designed for microbiology. In particular, the automation process has been applied to all the pre-analytical steps and to the evaluation of the results by using sophisticated artificial intelligence algorithms. The complete clinical laboratory automation is currently the main organizational challenge for microbiologists [50].

**Question 7. How can you manage the patient with infection/colonization of multidrug-resistant organisms (MDROs)?**

**Statement 7.1. The application of contact isolation precautions is always recommended for patients known or highly suspected for MDROs.**

MDROs including methicillin-resistant *Staphylococcus aureus* (MRSA), vancomycin-resistant *Enterococcus* (VRE), extended-spectrum β-lactamase (ESBL) producers, and *Klebsiella pneumoniae*-producing carbapenemase (KPC) pose significant public health challenges. The prevention and control of MDROs are a global priority.

Traditionally, hospitals have been considered the main reservoir of MDROs. Around 20–40% of nosocomial infections can be mainly attributed to cross-infection via the hands of healthcare personnel. Less frequently, patients can become colonized with nosocomial pathogens by direct contact with contaminated patient care equipment or contaminated surfaces in the healthcare environment [51].

Current strategies to address MDROs consist of the three following strategies [52]:

- Developing new antimicrobial agents
- Increasing antimicrobial stewardship efforts
- Interrupting MDROs cross-transmission

Bacteria tend to inhabit specific sites on either in the human body or in the hospital environment which serve as reservoirs for transmission. The reservoirs of resistant organisms include niches in the human microbiome. The microbiota of the skin, respiratory epithelium, and the gastrointestinal tract are altered within a few days in the hospital. Patients’ flora can be deranged by antibiotics, chemotherapy, or acquisition of nosocomial organisms. Patients who are colonized with resistant bacteria serve inadvertently as potential reservoirs for transmission. Colonization pressure, or the proportion of patients in a given unit who are colonized with resistant bacteria, is an independent risk factor for transmission. Surveillance cultures for carbapenem-resistant *Enterobacteriaceae* (CRE) have been advocated in a number of reports and recommendations as part of an overall strategy to combat it. Active screening for CRE using rectal surveillance cultures has been shown to be highly effective, when part of a comprehensive infection control program, in halting the spread of CRE in healthcare facilities [53].

Isolation or cohorting of colonized/infected patients is a cornerstone of IPC. Its purpose is to prevent the transmission of microorganisms from infected or colonized patients to other patients, hospital visitors, and HCWs, who may subsequently transmit them to other patients or become infected or colonized themselves. Isolating a patient with highly resistant bacteria is beneficial in stopping the patient-to-patient spread. Isolation measures should be an integral part of any IPC program; however, they are often not applied consistently and rigorously, because they are expensive, time-consuming, and often uncomfortable for patients.

Facilities should have written policies and procedures that identify patients with MDROs and should require that contact precautions are implemented in all practice settings. Communication is a vital component for successful implementation of contact precautions and must occur at all points in the perioperative process.

**Question 8. Antimicrobial stewardship: Is a multidisciplinary approach necessary?**

**Statement 8.1. The three basic requirements of an Antimicrobial Stewardship program are:**

- **The existence of a multidisciplinary antimicrobial stewardship team.**
- **A microbiological report on a fixed basis on the bacterial resistance of the hospital at least annually, if possible stratified by departments or at least for some key departments (e.g. Intensive Care Units).**
- **A report on fixed consumption of antibiotics in the hospital.**

**Statement 8.2. A multidisciplinary antimicrobial stewardship team should be coordinated by an infectious disease specialist, or by another specialist with documented infectious skills.**

Every hospital worldwide should utilize the existing resources to create an effective multidisciplinary team for combating AMR. The best strategies for combating AMR are not definitively established and are likely to vary based on local culture, policy, organization, and routine clinical practice despite several guidelines on the topic [44].

We propose that the best means of improving programs to contain AMR should involve collaboration among various specialties within a healthcare institution. They should focus on collaboration between all healthcare professionals to shared knowledge and widespread diffusion of practice. The involvement of HCWs may raise their awareness of AMR [44]. It is essential for any team to have at least one member who is an infectious disease specialist. Pharmacists with advanced training or long-standing clinical experience in infectious diseases are also key actors for the design and implementation of the stewardship program interventions monitoring consumption data of antibiotics. In any healthcare setting, a significant amount of energy should be spent on IPC. Infection control specialists and hospital epidemiologists should be always included in these programs to coordinate efforts on monitoring and preventing HAIs. Microbiologists should actively guide the proper use of tests and the flow of laboratory results. Being involved in providing surveillance data on AMR, they should provide periodic reports on AMR data allowing the multidisciplinary team to determine the ongoing burden of AMR in the hospital. Moreover, timely and accurate reporting of microbiology susceptibility test results allows the selection of more appropriate targeted therapy and may help reduce broad-spectrum antimicrobial use. Surgeons with adequate knowledge in surgical infections and surgical anatomy when involved may audit both antibiotic prescriptions and prevention practices, provide feedback to the prescribers and integrate best practices of antimicrobial use among surgeons, and act as champions among colleagues implementing change within their own sphere of influence. Infections are the main factors contributing to mortality in ICUs. Intensivists have a critical role in treating multidrug-resistant organisms in ICUs in critically ill patients. They have a crucial role in prescribing antimicrobial agents for the most challenging patients and are at the forefront of successful antibiotic prescribing policies. Emergency departments (EDs) represent a particularly important setting for addressing inappropriate antimicrobial prescribing practices, given the frequent use of antibiotics in this setting that sits at the interface of the community and the hospital. Therefore, also ED practitioners should be involved. Without adequate support from hospital management, these programs will be inadequate or inconsistent since the programs do not generate revenue. Engagement of hospital management has been confirmed as a key factor for both developing and sustaining. Finally, an essential participant who has been often unrecognized and underutilized is the “staff nurse” as nurses perform numerous functions that are integral to success.

**Question 9. Why and how do you have to monitor antibiotics consumption in the hospitals?**

**Statement 9.1. It is important to monitor antibiotics consumption. The data of the consumption data of antibiotics should be expressed in specific reports in defined daily doses.**

Pharmacy’s contribution to ASPs has significantly evolved over the course of the twenty-first century. Although microbiologists and infection specialist physicians have been conventionally responsible for providing advice on clinical management of infected patients, many pharmacists in clinical practice have now established roles complementing the expertise in multidisciplinary antimicrobial stewardship teams.

Pharmacists’ responsibilities for antimicrobial stewardship include promoting the optimal use of antimicrobial agents. Typical interventions include patient-specific recommendations on therapy; the implementation of policies, education, therapeutic drug monitoring, and participation in antimicrobial stewardship ward rounds [54, 55].

Antibiotics are prescribed in up to a third of hospital inpatients, often inappropriately [56], and more than two thirds of critically ill patients are on antibiotics at any given time during their hospital admission [57]. Antibiotic use is one of the most important parameters for assessing the impact that an ASP has on a hospital and its patient population, although microbiological resistance and clinical outcomes are also important measures. Antimicrobial measures looking at consumption are the most commonly used measures and are focused on defined daily dose (DDD), usually standardized per 1000 patient-days.

DDD is a metric that was developed in the 1970s and has been further refined and promoted by the WHO Collaborating Centre for Drug Statistics Methodology. DDD is described as “the assumed average maintenance dose per day for a drug used for its main indication in adults” [58]. In simple terms, a DDD is the amount of drug that a typical patient might receive on any day for therapeutic purposes. An important advantage of using DDDs is the relative ease of hospital systems to report consumption using DDDs: most pharmacy departments have a mechanism to calculate overall prescription, dispensing, or consumption of a quantity of antimicrobials, allowing DDDs/1000 patient-days to be relatively easy to calculate if bed utilization is also available. Additionally, institution-wide consumption can be benchmarked against similar institutions. The landmark guidelines on antimicrobial stewardship by the Infectious Diseases Society of America and Society for Healthcare Epidemiology of America advocated for DDDs/1000 patient-days as a universal metric for hospital-based ASPs [59].

**Question 10. What is the role for the nurse in preventing HAIs?**

**Statement 10.1. The nurse is an integral part of the multidisciplinary team for the prevention of infections across the surgical pathway.**

**Statement 10.2. It is important to implement educational and training interventions concerning the prevention of SSIs by following a modality appropriate to the level of education of the patient/caregiver.**

The role of the professional nurse in preventing HAIs is significant. The nurse is a member of the healthcare team who leads the rest of the team in practicing prevention strategies to protect the patient from infection [60]. Some of the most basic strategies resulting in positive patient outcomes include the following:

- The practice and promotion of hand hygiene
- Consistent use of aseptic technique
- Cleaning and disinfection practices
- Use of standard precautions
- Patient assessment and additional precautions
- Patient education
- Use of safety devices
- Removal of unnecessary invasive devices
- Use of bundle strategies for infection prevention
- Fit for duty

Nurses play a pivotal role in preventing hospital-acquired infections (HAIs), not only by ensuring that all aspects of their nursing practice are evidence-based, but also through patient education. One of the most important roles nurses have today is patient education. This was once reserved for the physician, but no longer. Today nurses assume more and more responsibility for educating patients and helping them to become responsible for their own health status. Patients need to take a proactive role in their own healthcare. This means they need to comprehend their health status and work to stabilize and prevent or minimize complications such as HAIs. Demographic variables, such as formal education level, reading ability, and barriers to participation in education, must be considered to maximize the effectiveness of self-management education outcomes. Hospital nurses can best educate patients by understanding that discharge planning begins with admission. Nurses have to ensure patients are effectively educated throughout their hospitalization so that they are prepared to care for themselves and participate in the care pathway.

**Question 11. Which are the principles of antibiotic prophylaxis?**

**Statement 11.1. Prolonging antibiotic prophylaxis after surgery is generally not associated with better clinical results.**

**Statement 11.2. There is no universally recognized intervention for improving the appropriateness of antibiotic prophylaxis in surgery. These interventions must be tailored to the type of surgeon and team to which they are addressed.**

Preoperative antibiotic prophylaxis (PAP) has been demonstrated in multiple randomized controlled trials and meta-analyses to reduce the risk of SSIs across different types of surgical procedures [61].

Given the evidence, systemic PAP is considered to be a key component of perioperative infection prevention bundles [62]. Although compliance with appropriate timing and spectrum of PAP has improved as a result of quality improvement initiatives, there remain significant deficiencies in compliance with other aspects of PAP such as duration of postoperative antibiotics [63, 64]. Given that approximately 15% of all antibiotics in hospitals are prescribed for surgical prophylaxis [65, 66], perioperative antibiotic prescribing patterns can be a major driver of some emerging infections (such as *C. difficile*) [67, 68] and selection of antibiotic resistance, increasing healthcare costs.

Although appropriate PAP plays a pivotal role in reducing the rate of SSIs [69], other factors that impact SSI rates should not be ignored. PAP should never substitute for good medical practices, such as those of IPC. Perioperative SSI prevention strategies should include attention to basic IPC strategies, surgical technique, hospital and operating room environments, instrument sterilization processes, and perioperative optimization of patient risk factors [70].

The key elements of appropriate surgical antimicrobial prophylaxis prescribing include the correct antimicrobial indication, drug dose, route, the timing of administration, and duration.

Joint guidelines for PAP in surgery were revised and updated in 2013 by the American Society of Health-System Pharmacists, Infectious Diseases Society of America, Surgical Infection Society, and Society for Healthcare Epidemiology of America [68]. These guidelines focus on the effective and safe use of AP. Therapeutic serum and tissue concentrations of antimicrobial agents should be present during the period of potential contamination. Additional antibiotic doses may need to be administered intraoperatively for prolonged procedures or for agents with short half-lives. In order to be safe, PAP should have no or few adverse effects and should have the narrowest spectrum of activity necessary to prevent postoperative infections.

There is no evidence that prolonging PAP after surgery can reduce the risk of SSIs. A single preoperative dose is adequate for the majority of procedures. Post-procedural doses of intravenous antibiotics (up to 24 h) may be only required in defined circumstances, such as some cardiac and vascular surgeries.

Below, seven practices for a correct surgical antibiotic prophylaxis are illustrated [71]:

1. Antibiotics alone are unable to prevent SSIs. Strategies to prevent SSIs should always include attention to the following:
  - IPC strategies including correct and compliant hand hygiene practices
  - Meticulous surgical techniques and minimization of tissue trauma
  - Hospital and operating room environments
  - Instrument sterilization processes
  - Perioperative optimization of patient risk factors
  - Perioperative temperature, fluid, and oxygenation management
  - Targeted glycemic control
  - Appropriate management of surgical wounds
2. Antibiotic prophylaxis should be administered for operative procedures that have a high rate of postoperative SSI, or when foreign materials are implanted.
3. Antibiotics given as prophylaxis should be effective against the aerobic and anaerobic pathogens most likely to contaminate the surgical site, i.e., Gram-positive skin commensals or normal flora colonizing the incised mucosae.
4. Antibiotic prophylaxis should be administered within 120 min prior to the incision. However, administration of the first dose of antibiotics beginning within 30–60 min before the surgical incision is recommended for most antibiotics (e.g., cefazolin), to ensure adequate serum and tissue concentrations during the period of potential contamination. Obese patients ≥ 120 kg require higher doses of antibiotics.
5. A single dose is generally sufficient. Additional antibiotic doses should be administered intraoperatively for procedures > 2–4 h (typically where duration exceeds two half-lives of the antibiotic) or with associated significant blood loss (> 1.5 L).
6. There is no evidence to support the use of postoperative antibiotic prophylaxis.
7. Each institution is encouraged to develop guidelines for the proper surgical prophylaxis.

Knox and Edye [63] demonstrated that an educational ASP was ineffective in changing surgical prophylactic antibiotic prescribing in an Australian hospital. Although that study was disappointing as far as showing improved behaviors, others have shown that ASPs may have a significant impact on optimizing antibiotic use in surgical prophylaxis practices [72, 73]. Van Kasteren et al. [73] in a prospective multisite study of elective procedures in 13 Dutch hospitals evaluated the quality of prophylaxis auditing before and after an intervention consisting of performance feedback and implementation of national clinical practice guidelines. Antimicrobial use decreased from 121 to 79 DDD/100 procedures, and costs reduced by 25% per procedure. After the intervention, antibiotic choice was inappropriate in only 37.5% of the cases instead of 93.5% expected cases in the absence of any intervention. Prolonged prophylaxis was observed in 31.4% instead of 46.8% expected cases and inappropriate timing in 39.4% instead of the expected 51.8%. Time series analysis showed that all improvements were statistically significant (*P* < 0.01). The overall SSI rates before and after the intervention were 5.4% (95% CI 4.3–6.5) and 4.6% (95% CI 3.6–5.4), respectively [73]. Huh et al. [72] performed an interrupted time-series study of an ASP relating to surgical prophylaxis in a tertiary care hospital. The ASP consisted of monitoring of performance indicators and implementation of a computerized decision support system. The program was effective in improving multiple measures including the total use of antibiotics, the use of third-generation cephalosporins and aminoglycosides, trends in proportions of resistant bacterial strains such as meropenem-resistant *Pseudomonas aeruginosa*, and length of stay. Saied et al. [12] implemented ASPs in 5 tertiary, acute-care surgical hospitals. The ASPs consisted of education aimed at surgeons and anesthesiologists, audit and feedback, and selection of surgeon champions. The efficacy of the intervention on timing and duration of antibiotic prophylaxis varied across hospitals when measured pre- and post-ASP implementation. Local factors such as available resources and stakeholder engagement likely play a role in the conflicting results of ASPs addressing surgical prophylaxis across different settings, as seen in these studies.

**Question 12. Which are the principles of antibiotic therapy?**

**Statement 12.1. It is important to know the local epidemiological context to define therapeutic protocols / guidelines for surgical infections treatment.**

**Statement 12.2. It is important to frame clinical conditions, in particular to differentiate between critical and non-critical patients.**

**Statement 12.3. It is important to pursue as much as possible targeted therapy or in any case a de-escalation in order to preserve some molecules: e.g.: carbapenems.**

**Statement 12.4. It is important to assess properly the duration of therapy based on source control.**

**Statement 12.5. In the setting of uncomplicated intra-abdominal infections including uncomplicated acute cholecystitis and acute appendicitis post-operative antimicrobial therapy is not necessary.**

**Statement 12.6. In patients with complicated intra-abdominal infections, when patients are not severely ill and when source control is complete, a short course (3-5 days) of post-operative therapy is suggested.**

**Statement 12.7. In patients with ongoing or persistent intra-abdominal infections, the decision to continue, revise, or stop antimicrobial therapy should be made on the basis of clinician judgment and laboratory information.**

Empirical antibiotic therapy should be based on local epidemiology, individual patient risk factors for difficult-to-treat pathogens, clinical severity of infection, and infection source. Initial antibiotic therapy for surgical infections is empirical in nature because microbiological data (culture and susceptibility results) may require > 24/48 h before they are available for a more detailed analysis. However, the results direct expansion of antimicrobial regimen if it is too narrow and perform a de-escalation if it is too broad [74, 75], particularly in critically ill patients where de-escalation strategy is one of the cornerstones of ASPs [76]. The principles of empiric antibiotic treatment should be defined according to the most frequently isolated microbes, always taking into consideration the local trend of antibiotic resistance. In this era of prevalent drug-resistant microorganisms, the threat of resistance is a source of major concern that cannot be ignored [76].

In the past 20 years, the incidence of intra-abdominal infections (IAIs) caused by MDROs has risen dramatically [76]. Quinolone resistance, prevalence of ESBL-producing bacteria, prevalence and mechanisms of carbapenem resistance in the local environment, and the place of recent traveling should be always taken into account when antibiotic therapy is administered empirically. Generally, the most important factors in predicting the presence of resistant pathogens are acquisition in a healthcare setting (particularly if the patient becomes infected in the ICU or has been hospitalized for more than 1 week), corticosteroid use, organ transplantation, baseline pulmonary or hepatic disease, and previous antimicrobial therapy [76, 77].

Previous antibiotic therapy is one of the most important risk factors for resistant pathogens [78]. Inappropriate therapy in critically ill patients may have a strong negative impact on the outcome. An ineffective or inadequate antimicrobial regimen is one of the variables more strongly associated with unfavorable outcomes in critically ill patients. Broad empiric antibiotic therapy should be started as soon as possible in patients with organ dysfunction (sepsis) and septic shock [79–83]. International guidelines for the management of sepsis and septic shock (the Surviving Sepsis Campaign) recommend intravenously administered antibiotics as soon as possible and in any case within the first hour of onset of sepsis and the use of broad-spectrum agents with adequate penetration of the presumed site of infection [84].

The results of microbiological testing may have great importance for the choice of therapeutic strategy of every patient, in particular, in the adaptation of targeted antibiotic treatment. They provide an opportunity to expand the antibiotic regimen if the initial choice was too narrow but also allow de-escalation of antibiotic therapy if the empirical regimen was too broad. Antibiotic de-escalation has been associated with lower mortality rates in ICU patients and is now considered a key practice for antimicrobial stewardship purposes [75]. The duration of antibiotic therapy has been studied appropriately in the setting of intra-abdominal infections (IAIs).

In the event of uncomplicated IAIs, the infection involves a single organ and does not extend to the peritoneum. When the source of infection is treated effectively by surgical excision, postoperative antibiotic therapy is not necessary, as demonstrated in managing uncomplicated acute appendicitis or cholecystitis [85–87].

In 2015, an important prospective study on the appropriate duration of antibiotic therapy in patients with complicated IAIs was published [88]. The study randomized 518 patients with complicated IAIs and adequate source control to receive antibiotics until 2 days after the resolution of fever, leukocytosis, and ileus, with a maximum of 10 days of therapy (control group), or to receive a fixed course of antibiotics (experimental group) for 4 ± 1 calendar days. In patients with complicated IAIs who had undergone an adequate source control procedure, the outcomes after fixed-duration antibiotic therapy (approximately 4 days) were similar to those after a long course of antibiotics (approximately 8 days) that extended until after the resolution of physiological abnormalities. In this study, most patients were not severely ill.

The high mortality associated with abdominal sepsis requires clinicians to maintain a high index of clinical suspicion of treatment failure and the early diagnosis of ongoing infections. These patients should always be monitored carefully including the potential use of inflammatory response markers.

Below, we report 13 practices in an appropriate antibiotic therapy across the surgical pathway [71]:

1. The source of infection should always be identified and controlled as soon as possible.
2. Antibiotic empiric therapy should be initiated after a treatable surgical infection has been recognized, since microbiological data (culture and susceptibility results) may not be available for up to 48–72 h to guide targeted therapy.
3. In critically ill patients, empiric broad-spectrum therapy to cover all likely pathogens should be initiated as soon as possible after a surgical infection has been recognized**.** Empiric antimicrobial therapy should be narrowed once culture and susceptibility results are available and/or adequate clinical improvement is noted.
4. Empirical therapy should be chosen on the basis of local epidemiology, individual patient risk factors for MDR bacteria and *Candida* spp., clinical severity, and infection source.
5. Specimens for microbiological evaluation from the site of infection are always recommended for patients with hospital-acquired or with community-acquired infections at risk for resistant pathogens (e.g., previous antimicrobial therapy, prior infection or colonization with a multidrug-resistant, extensively drug-resistant, and pan-drug-resistant pathogens) and in critically ill patients. Blood cultures should be performed before the administration of antibiotics in critically ill patients.
6. Antibiotics dose should be optimized to ensure that pharmacokinetic-pharmacodynamic (PK-PD) targets are achieved. This involves prescribing of an adequate dose, according to the most appropriate and right method and schedule to maximize the probability of target attainment.
7. The appropriateness and need for antimicrobial treatment should be reassessed daily.
8. Once source control is established, short courses of antibiotic therapy are as effective as longer courses regardless of signs of inflammation.
  - Intra-abdominal infection: 4 days are as effective as 8 days in moderately ill patients.
  - Bloodstream infection: 5–7 days are as effective as 7–21 days for most patients.
  - Ventilator-associated pneumonia: 8 days are as effective as 15 days.
9. Failure of antibiotic therapy in patients having continued evidence of active infection may require a re-operation for a second source control intervention.
10. Biomarkers such as procalcitonin (PCT) may be useful to guide duration and/or cessation of antibiotic therapy in critically ill patients.
11. Clinicians with advanced training and clinical experience in surgical infections should be included in the care of patients with severe infections.
12. IPC measures, combined with ASPs, should be implemented in surgical departments. These interventions and programs require regular, systematic monitoring to assess compliance and efficacy.
13. Monitoring of antibiotic consumption should be implemented and feedback provided to all ASP team members regularly (e.g., every 3–6 months) along with resistance surveillance data and outcome measures.

**Question 13. How can you manage invasive candidiasis in surgical patients?**

**Statement 13.1. It is important the knowledge of the risk of developing invasive candidiasis, improve microbiological diagnostics and optimize treatment**.

Invasive candidiasis (IC) has a significant impact on morbidity, mortality, length of hospital stay, and healthcare costs in critically ill patients [89]. The overall mortality for these patients is high. Candidemia increases mortality rates in the range of 20–49% [90], and the attributable mortality has been calculated to be around 15% [91]. The severity of illness (APACHE II score > 10, ventilator use for > 48 h), antibiotics, central venous lines, total parenteral nutrition, burns, and immunosuppression are the most common risk factors [92].

The risk factors for IC are so numerous that most patients could be considered as exhibiting risk factors for IC. But, the use of excessive antifungal agents would be associated with substantially increased overall healthcare costs and might lead to the emergence of resistance. Unfortunately, early diagnosis of IC remains a challenge, and criteria for starting empirical antifungal therapy in ICU patients are poorly defined. To both ensure appropriate and timely antifungal therapy and to avoid unnecessary use of antifungal agents, some authors have developed clinical prediction rules to identify patients at high risk of candidiasis and for whom initiation of empirical antifungal therapy could be justified. However, there are many concerns about these rules: high specificity but low sensitivity. In 2006, a Spanish group, using the database of the Estudio de Prevalencia de CANdidiasis project, identified four predictors of proven invasive *Candida* infection. Based on these predictors, a score named “Candida score” (CS) was built. In 2009, the same group demonstrated a significant linear association between increasing values of the CS and the rate of invasive *Candida* infections [93]. The factors to predict IC were surgery, multifocal colonization, total parenteral nutrition, and severe sepsis. To each risk factor, 1 point was given, and for clinical sepsis, a score of 2 was given. The cutoff value of 2.5 had sensitivity of 81% and specificity of 74% [93]. Although blood cultures are still considered the gold standard for diagnosis, it has been shown that they are negative in up to 50% of cases [94]. Thus, non-culture diagnostic techniques based on serological biomarkers detecting fungal cell components and/or antibodies directed against these components have been investigated. All these diagnostic tests may diagnose IC earlier than clinical or culture-based measures.

Among the biomarkers, mannan antigen and antigen-antibody complex showed higher sensitivity and specificity when combined together [95]. In a meta-analysis of 14 studies, 7 of which were performed in non-neutropenic critically ill patients; the sensitivity and specificity of mannan and anti-mannan IgG were 58% and 93%, and 59% and 83%, respectively. Values for the combined assay were 83 and 86%, with the best performances for *C. albicans*, *C. glabrata*, and *C. tropicalis* infection [95]. The 1,3-beta-d-glucan (BG) is a fungal cell wall antigen that can be detected in the blood of patients with a sensitivity of 56–93% and a specificity of 71–100% for IC [96]. Thanks to its high negative predictive value, BG is potentially useful for the therapy decision-making process and discontinuing of empirical antifungal therapy. An integrated strategy with BG and CS helped to withhold or discontinue treatment, saving health costs without increasing mortality in 198 severely ill patients admitted to ICU with sepsis and a CS > 3 [97]. Once the diagnosis is made, early systemic treatment is warranted. The armamentarium of drugs for the treatment of candidiasis currently comprises 3 major drug classes: the polyenes, azoles, and echinocandins.

The majority of patients with candidemia have indwelling CVCs when the diagnostic blood culture is obtained [98], but differentiating between CVC- and non-CVC-related candidemia is not always straightforward. *C. parapsilosis* is particularly frequent as a cause of CVC infection. There is compelling evidence that CVC removal is associated with higher rates of treatment success and lower mortality rates as compared with CVC retention [98]. Despite contradictory data from a post hoc analysis of 2 clinical trials [98], it is generally accepted that indwelling CVCs should be removed as early as possible in all patients with candidemia [99]. CVCs should be urgently removed in patients with septic shock. For clinically stable patients for whom immediate CVC removal presents significant difficulties, for example, due to limited vascular access, establishing a diagnosis of CVC infection may be of importance.

**Question 14. Which are the principles of antibiotic therapy in critically ill patients?**

**Statement 14.1. In critically ill patients, antibiotic therapy should be prescribed using a severity and risk stratification driven approach.**

**Statement 14.2. It is important to support the need for better identification of patients at risk of MDROs infection, more accurate diagnostic tools enabling a rule-in/rule-out approach for bacterial sepsis, the use of adequate dosing and administration schemes to ensure the attainment of pharmacokinetics/pharmacodynamics targets, concomitant source control when appropriate, and a systematic reappraisal of initial therapy in an attempt to minimize collateral damage on commensal ecosystems through de-escalation and treatment-shortening whenever conceivable.**

The rapid global spread of multiresistant bacteria and loss of antibiotic effectiveness increases the risk of initial inappropriate antibiotic therapy (IAT) and poses a serious threat to patient safety especially in critically ill patients. A systematic review and meta-analysis of published studies to summarize the effect of appropriate antibiotic therapy or IAT against Gram-negative bacterial infections in the hospital setting was published in 2014 [100]. Using a large set of studies, the authors found that IAT is associated with a number of serious consequences, including an increased risk of hospital mortality.

Infections caused by drug-resistant, Gram-negative organisms represent a considerable financial burden to healthcare systems due to the increased costs associated with the resources required to manage the infection, particularly longer hospital stays. However, given the impact of early and broad-spectrum empirical therapy and the emphasis on this in international guidelines, there is a low threshold for initiating antibiotics in many patients with suspected infection. This has led to the widespread use of antibiotics in critically ill patients, which is often unnecessary or inappropriate [101].

The massive consumption of antibiotics in the ICU is responsible for substantial ecological side effects that promote the dissemination of MDROs. Strikingly, up to half of ICU patients receiving empirical antibiotic therapy have no definitively confirmed infection, while de-escalation and shortened treatment duration are insufficiently considered in those with documented sepsis.

Published data notably support [102] the following:

- The need for better identification of patients at risk of MDROs infection
- More accurate diagnostic tools enabling a rule-in/rule-out approach for bacterial sepsis
- An individualized reasoning for the selection of single-drug or combination empirical regimen
- The use of adequate dosing and administration schemes to ensure the attainment of PK-PD targets
- Concomitant source control when appropriate
- A systematic reappraisal of initial therapy in an attempt to minimize collateral damage on commensal ecosystems through de-escalation and treatment shortening whenever conceivable

Several trials found PCT-guided antibiotic stewardship to reduce antibiotic exposure and associated side effects among patients with respiratory infection and sepsis [103]. Decisions regarding antibiotic use in an individual patient are complex and should be based on the pre-test probability for bacterial infection, the severity of presentation, and the results of the PCT. In the context of a low pre-test probability for bacterial infections and a low-risk patient, a low PCT level helps to rule out bacterial infection and empiric antibiotic therapy can be avoided. In the context of a high pre-test probability for bacterial infections and/or a high-risk patient with sepsis, monitoring of PCT over time helps to track the resolution of infection and decisions regarding the early stop of antibiotic treatment. Although these concepts have been successful in several respiratory infection and sepsis trials, some studies failed to show an added benefit of PCT due to factors such as low protocol adherence and relying on single rather than repeat PCT measurements.

In this era of AMR, another interesting strategy is a therapeutic approach based on patient risk stratification. Especially for Gram-negative MDRO infections, an approach based on the patient risk stratification could improve outcomes and avoid antibiotic misuse.

This approach could help physicians to avoid antibiotic overuse as well as to start promptly with the most appropriate antibiotic regimen. Several risk factors for Gram-negative MDRO infections have been identified. These include prior infection or colonization with Gram-negative MDROs, antibiotic therapy in the past 90 days, poor functional status performance, hospitalization for more than 2 days in the past 90 days, occurrence five or more days after admission to an acute hospital, receiving hemodialysis, and immunosuppression [104]. Moreover, prior receipt of carbapenems, broad-spectrum cephalosporins, and fluoroquinolones has been associated specifically with MDR *Pseudomonas aeruginosa* [105].

Recently, the high mortality and mortality associated with multidrug-resistant Gram-negative bacteria along with limited treatment options have led to a resurgence in the use of the nephrotoxic drug colistin. Fortunately, several new antibiotic agents with activity against Gram-negative MDROs, including ceftazidime/avibactam and ceftolozane/tazobactam, have become available. Further studies are needed to elucidate their place in therapy and their impact on real-world outcomes such as length of stay and mortality, especially for ICU patients; however, these are the few resources we have and should not be wasted unnecessarily.

**Question 15. Who is the surgeon champion?**

**Statement 15.1. To be a champion in preventing and managing infections in surgery means to create a culture of collaboration in which infection prevention, antimicrobial stewardship and correct surgical approach are of high importance.**

There is sometimes a false impression that HAIs are adequately controlled. However, with multidrug-resistant bacteria increasing, such as MRSA, VRE, carbapenem-resistant *Enterobacteriaceae* (CRE), such infections are more than ever a public health threat. It is well known that HAIs tend to show higher resistance rates to antibiotics than community-acquired infections.

Patients in hospitals are often exposed to multiple risk factors for the acquisition of multidrug-resistant bacteria. Acute care facilities are important sites for the development of AMR. The intensity of care together with populations highly susceptible to infection creates an environment that facilitates both the emergence and transmission of resistant organisms.

Surgeons with satisfactory knowledge in surgical infections involved in both IPC team and in the ASPs may integrate the best practice among surgeons. Although the surgeon’s impact on the incidence of SSIs has not yet been examined in a comprehensive manner, some reports have reported that the incidence of SSIs varies widely between hospitals and between surgeons [106, 107], suggesting that working practices play a critical role in the prevention of these infections and that more may be done to improve infection control in routine surgical practice.

Very few studies have focused on the relationship between ASPs and surgeons. In 2015, Çakmakçi [108] suggested that the engagement of surgeons in ASPs might be crucial to their success. However, in 2013, Duane et al. [109] showed poor compliance with surgical services with ASP recommendations. A retrospective study by Sartelli et al. [110] showed that implementation of an education-based ASP achieved a significant improvement in all antimicrobial agent prescriptions and a reduction in antimicrobial drug consumption. In a surgical unit performing mainly elective major abdominal surgery and emergency surgery, both a local protocol of surgical prophylaxis and a set of guidelines for the management of intra-abdominal infections (IAIs) and control of antimicrobial agent use were introduced. Comparing the pre-intervention and post-intervention periods, the mean total monthly antimicrobial use decreased by 18.8%, from 1074.9 defined DDDs per 1000 patient-days to 873.0 DDDs per 1000 patient-days after the intervention. The model was based on the concept of the “surgeon champion.” The “champion” was a surgeon who on a day-to-day basis worked within the surgical unit, promoting and maintaining a culture in which IPC appropriate use of antibiotics was of high importance. Identifying a local opinion leader to serve as a champion may be important because the “surgeon champion” may integrate the best clinical practices and drive the colleagues in changing behaviors. We believe that the concept of the “surgeon champion” can be a crucial way to improve IPC across the surgical pathway.

**Question 16. Which are the principles of source control?**

**Statement 16.1. The timing and adequacy of source control are important in the management of surgical infections; late and/or incomplete procedures may have severely adverse consequences on outcome especially in critically ill patients.**

Source control encompasses all measures undertaken to eliminate the source of infection, reduce the bacterial inoculum, and correct or control anatomic derangements to restore normal physiologic function [111, 112].

As a general principle, every verified source of infection should be controlled as soon as possible. The level of urgency of treatment is determined by the affected organs, the relative speed at which clinical symptoms progress and worsen, and the underlying physiological stability of the patient. Non-operative interventional procedures include percutaneous drainages of abscesses. Ultrasound- and CT-guided percutaneous drainage of abdominal and extraperitoneal abscesses in selected patients are safe and effective. The principal cause for failure of percutaneous drainage is misdiagnosis of the magnitude, extent, complexity, and location of the abscess [113].

Surgery is the most important therapeutic measure to control surgical infections. In the setting of intra-abdominal infections, the primary objectives of surgical intervention include determining the cause of peritonitis, draining fluid collections, and controlling the origin of the abdominal sepsis. In patients with intra-abdominal infections, surgical source control entails resection or suture of a diseased or perforated viscus (e.g., diverticular perforation, gastro-duodenal perforation), removal of the infected organ (e.g., appendix, gallbladder), debridement of necrotic tissue, resection of ischemic bowel, and repair/resection of traumatic perforations with primary anastomosis or exteriorization of the bowel.

In certain circumstances, infection not completely controlled may trigger an excessive immune response and local infection may progressively evolve into sepsis, septic shock, and organ failure. Such patients can benefit from immediate and aggressive surgical re-operations with subsequent re-laparotomy strategies, to curb the spread of organ dysfunctions caused by ongoing peritonitis. Surgical strategies following an initial emergency laparotomy include subsequent “re-laparotomy on demand” (when required by the patient’s clinical condition) as well as planned re-laparotomy in the 36–48h postoperative period [114].

On-demand laparotomy should be performed only when absolutely necessary and only for those patients who would clearly benefit from additional surgery. Planned re-laparotomies, on the other hand, are performed every 36–48 h for purposes of inspection, drainage, and peritoneal lavage of the abdominal cavity. The concept of a planned re-laparotomy for severe peritonitis has been debated for over 30 years. Re-operations are performed every 48 h for reassessing the peritoneal inflammatory process until the abdomen is free of ongoing peritonitis; then the abdomen is closed. The advantages of the planned re-laparotomy approach are optimization of resource utilization and reduction of the potential risk for gastrointestinal fistulas and delayed hernias. The results of a clinical trial published in 2007 by Van Ruler et al. [115] investigating the differences between on-demand and planned re-laparotomy strategies in patients with severe peritonitis found few advantages for the planned re-laparotomy strategy; however, the study mentioned that this latter group exhibited a reduced need for additional re-laparotomies, decreased patient dependency on subsequent healthcare services, and decreased overall medical costs.

An open abdomen (OA) procedure is the best way of implementing re-laparotomies. Open abdomen (OA) procedure is defined as intentionally leaving the fascial edges of the abdomen unapproximated (laparostomy). The abdominal contents are exposed and protected with a temporary coverage. The OA technique, when used appropriately, may be useful in the management of surgical patients with severe abdominal sepsis [116]. However, the role of the OA in the management of severe peritonitis is still being debated. The role of the OA in the management of severe peritonitis has been a controversial issue [116]. Although guidelines suggest not to routinely utilize the open abdomen approach for patients with severe intra-peritoneal contamination undergoing emergency laparotomy for intra-abdominal sepsis, OA has now been accepted as a strategy in treating physiologically deranged patients with acute peritonitis.

**Question 17. What is the role of the biomarkers in surgery?**

**Statement 17.1. C-reactive protein (CRP) and procalcitonin (PCT) can help clinicians to diagnose surgical infections.**

**Statement 17.2. PCT can help clinicians in early discontinuation of antibiotics in critically ill patients and in patients undergoing intervention for acute peritonitis.**

Although more than a hundred biomarkers have been studied, only a limited number of them became routinely available in clinical practice. CRP and PCT are the more frequently studied and used biomarkers.

Serum CRP is an acute-phase protein synthesized exclusively in the liver. Its secretion is initiated 4 to 6 h after an inflammatory insult (effect mediated by cytokines namely interleukin-6), and its concentration doubles every 8 h with a peak at 36–50 h [117]. The sole determinant of CRP plasmatic levels is its synthesis rate, which is proportional to the intensity of the inflammatory insult. Its production and elimination are not influenced by renal replacement therapy or immunosuppression (both systemic steroids and neutropenia). It has a sensitivity of 68–92% and a specificity of 40–67% as a marker of bacterial infection. Its low specificity and inability to differentiate bacterial infections from non-infectious causes of inflammation make CRP of limited diagnostic value [118]. The available assays for CRP measurement are reliable, stable, reproducible, rapid, inexpensive, and present an acceptable limit of detection (0.3–5 mg/L). CRP has been analyzed in multiple clinical contexts, but only a small number of studies have focused on its use for optimizing antibiotic therapy [119]. In primary care, CRP improves the assessment of the severity of infection and extent of inflammation [120] and performs better in predicting the diagnosis of pneumonia than any individual or combination of clinical signs and symptoms. A Cochrane review [121] demonstrated reduced antibiotic prescription with CRP testing, which led to its incorporation in the National Institute for Health and Care Excellence (NICE) guidelines for the diagnosis of pneumonia. CRP has been reported to be useful in the diagnosis of appendicitis; however, it lacks specificity. Multiple studies have examined the sensitivity of CRP level alone for the diagnosis of appendicitis in patients selected to undergo appendectomy. Gurleyik et al. noted a CRP sensitivity of 96.6% in 87 of 90 patients with a histologically proven disease [122]. Similarly, Shakhatreh found a CRP sensitivity of 95.5% in 85 of 89 patients with histologically proven appendicitis [123]. Asfar et al. reported a CRP sensitivity of 93.6% in 78 patients undergoing appendectomy [124].

PCT is a precursor protein of calcitonin that can be produced ubiquitously throughout the body. It is released 3–4 h after an inflammatory stimulus with a plasmatic peak within 6–24 h and a half-life ranging from 22 to 35 h. Its plasmatic levels are markedly influenced by renal function, different techniques of renal replacement therapy, and neutropenia. It showed a sensitivity of 77% and a specificity of 79% for early diagnosis of sepsis in critically ill patients [117, 118]. PCT is the most widely studied biomarker for antibiotic stewardship. It has been tested as an aid to the initiation and/or discontinuation of antibiotics, both in children and adults presenting with distinct sources of infection and in different scenarios. Multiple trials have investigated the benefits of using serum PCT levels to guide whether and for how long antibiotic therapy is used—a process referred to as a PCT-guided antibiotic stewardship—in patients with infection in the ICU [125–135]. Several studies have demonstrated the benefits of using serum PCT levels to guide antibiotic therapy in patients undergoing intervention for acute peritonitis [136, 137].

**Question 18. Who are the patients at high risk for surgical site infections?**

**Statement 18.1. A number of risk factors are known to increase the incidence of SSIs, they can be effective at three different levels: patient, operative (surgical procedure-related) and institutional level (hospital related).**

**Statement 18.2. Although multiple strategies exist for identifying surgical patients at high risk for SSIs, no one strategy is superior for all patients and further efforts are necessary to determine if risk stratification in combination with risk modification can reduce SSIs in this patients’ population.**

SSIs are a significant healthcare quality issue, resulting in increased morbidity, disability, length of stay, mortality, resource utilization, and costs. Identification of high-risk patients may improve preoperative counseling, inform resource utilization, and allow modifications in perioperative management to optimize outcomes.

Many risk factors are beyond practitioner control, but optimizing perioperative conditions can certainly help decrease infection risk [138]. High-risk surgical patients may be identified on the basis of individual risk factors or combinations of factors. In particular, statistical models and risk calculators may be useful in predicting infectious risks, both in general and for SSIs. These models differ in the number of variables: inclusion of preoperative, intraoperative, or postoperative variables; ease of calculation; and specificity for particular procedures. Furthermore, the models differ in their accuracy in stratifying risk.

Although multiple strategies exist for identifying surgical patients at high risk for SSIs, no strategy is superior for all patients, and further efforts are necessary to determine if the risk stratification in combination with risk modification can reduce SSIs in this patient population [138].

Early evaluation of perioperative SSI risk factors and patient risk stratification could be of great value in the development of predictive risk models [139]. Predictive risk models could, in turn, assist surgeons and their patients in the clinical decision-making process (e.g., counseling patients on the appropriateness and risks of surgery). In addition, risk models could be used to develop targeted perioperative prevention strategies and diagnostic care process models and improve risk adjustment for risk modeling used in the public reporting of SSI as a quality metric [139].

However, a study reviewing SSIs in patients undergoing colorectal resections (C-SSIs), identified from an institutional ACS-NSQIP dataset (2006 to 2014), showed that published risk prediction models do not accurately predict C-SSI in their own independent institutional dataset [140]. Application of externally developed prediction models to any individual practice must be validated or modified to account for the institution and case-mix specific factors. This questions the validity of using externally or nationally developed models for “expected” outcomes and interhospital comparisons.

**Question 19. How can you care post-operative wounds to prevent surgical site infections?**

**Statement 19.1. Advanced dressing of any type should not be used for primarily closed surgical wounds for the purpose of preventing SSI.**

**Statement 19.2. The surgical wound dressing can be removed for a minimum of 48 hours after surgery unless leakage occurs. There is no evidence that extending medication time implies a reduction in SSIs.**

Appropriate surgical wound and incision management in the postoperative time period is imperative to prevent SSIs. The term “surgical wound” used in this document refers to a wound created when an incision is made with a scalpel or other sharp cutting device and then closed in the operating room by suture, staple, adhesive tape, or glue and resulting in a close approximation of the skin edges [141]. It is traditional to cover such wounds with a dressing, which acts as a physical barrier to protect the wound from contamination from the external environment until it becomes impermeable to microorganisms.

To assess the effects of wound dressings compared with no wound dressings, and the effects of alternative wound dressings, in preventing SSIs in surgical wound healing by primary intention, a Cochrane review was published in 2016 [142]. The authors concluded that it is uncertain whether covering surgical wound healing by primary intention with wound dressings reduces the risk of SSI, or whether any particular wound dressing is more effective than others in reducing the risk of SSI, improving scarring, reducing pain, improving acceptability to patients, or is easier to remove. Most studies in this review were small and at a high or unclear risk of bias. Based on the current evidence, decision-makers may wish to base decisions about how to dress a wound following surgery on dressing costs as well as patient preference.

The WHO Global Guidelines for the Prevention of SSIs [5, 6] suggest not using any type of advanced dressing over a standard dressing on primarily closed surgical wounds for the purpose of preventing SSI. Low-quality evidence from ten RCTs shows that advanced dressings applied on primarily closed incisional wounds do not significantly reduce SSI rates compared to standard wound dressings. Postoperative care bundles recommend that surgical dressings be kept undisturbed for a minimum of 48 h after surgery unless leakage occurs [143, 144].

**Question 20. How can we engage surgeons in appropriate infection prevention and management?**

**Statement 20.1. Active education techniques, such as academic detailing, consensus building sessions and educational workshops, should be implemented in each hospital worldwide according to its own resources.**

**Statement 20.2. Surgeons with satisfactory knowledge in surgical infections should be involved in the infection control team and recognized as “champions” by the hospital's administration.**

Surgeons should be involved in guideline development, and their implementation should translate practice recommendations into a protocol or pathway that specifies and coordinates responsibilities and timing for particular actions among a multidisciplinary team. Although both the WHO [5, 6] and CDC [7] have recently published guidelines for the prevention of SSIs, knowledge and awareness of IPC measures among surgeons are often inadequate and a great gap exists between the best evidence and clinical practice with regards to SSI prevention.

Education is crucial in improving HCW behaviors towards HAIs. Effective prevention and management of HAIs is a process requiring a fundamental understanding of the evolving relationship between inappropriate prevention and management and the prevalence of HAIs and the emergence of AMR. However, because medical professionals have already established their knowledge, attitudes, and behaviors, it is difficult to change their deeply established views and behaviors. Increasing knowledge may influence their perceptions and motivate them to change behavior. Education and training represent an important component for the accurate implementation of recommendations. Education of all health professionals in preventing HAIs should begin at the undergraduate level and be consolidated with further training throughout the postgraduate years. Hospitals are responsible for educating clinical staff about IPC programs. Active education techniques, such as academic detailing, consensus building sessions and educational workshops, should be implemented in each hospital worldwide according to its own resources. Efforts to improve educational programs are required, and it is necessary that every hospital worldwide develops appropriate educational programs to drive HCWs towards correct behaviors in the prevention and management of HAIs. The purpose of training and educating healthcare professionals should be to ensure both individual understanding and a team approach with shared knowledge, skills, and attitudes towards the prevention and management of HAIs.

Peer-to-peer role modeling and champions on an interpersonal level have been shown to positively influence the implementation of infection control practices. Many practitioners use educational materials or didactic continuing medical education sessions to keep up to date. However, these strategies might not be very effective in changing practice, unless education is interactive and continuous and includes discussion of evidence, local consensus, feedback on performance (by peers), and making personal and group learning plans. Identifying a local opinion leader to serve as a champion may be important because the “champion” may integrate best clinical practices and drive the colleagues in changing behaviors, working on a day-to-day basis, and promoting a culture in which IPC is of high importance. Surgeons with satisfactory knowledge in surgical infections may provide feedback to the prescribers, integrate the best practices among surgeons and implement changes within their own sphere of influence interacting directly with the IPC team.

Raising awareness of IPC to stakeholders is another crucial factor in changing behaviors. Probably, clinicians are more likely to comply with the guidelines when they have been involved in developing the recommendations. One way to engage health professionals in guideline development and implementation is to translate practice recommendations into a protocol or pathway that specifies and coordinates responsibilities and timing for particular actions among a multidisciplinary team.

## Conclusions

Leading international organizations, such as the WHO, acknowledge that collaborative practice is essential for achieving a concerted approach to providing care [1]. Prevention and management of infections across the surgical pathway should always focus on the collaboration between all healthcare professionals with shared knowledge and widespread diffusion of best practice. In the Appendix, the statements approved with an agreement ≥ 80% are reported.

## Data Availability

Not applicable.

## Appendix

Statements approved with an agreement ≥80%

*Question 1. How can you implement global guidelines for the prevention of surgical site infections (SSIs)?*

**Statement 1.1.** Recent global guidelines for the prevention of SSIs can support healthcare workers to develop or strengthen infection prevention and control programs, with a focus on surgical safety, as well as AMR action plans. All healthcare workers should adopt these evidence-based recommendations in their clinical practice.

**Statement 1.2.** A safer surgical care requires a range of precautions aimed at reducing the risk of SSIs before, during and after surgery.

**Statement 1.3.** To support local implementation of guidelines for the prevention of SSIs, 5 steps of a multimodal strategy, including system change, training and education, evaluation and feedback, communications for awareness raising and institutional safety climate and culture are suggested.

*Question 2. Why do you have to survey HAIs?*

**Statement 2.1**. Surveillance of HAIs improves the quality of care because it reduces the risk of infection. It should be supported by all healthcare workers.

*Question 3. How can you implement the prevention of HAIs?*

**Statement 3.1.** It is necessary to set up a solid and branched surveillance network gathering alert signals, verifying their severity and initiating the organizational response via “warnings”.

**Statement 3.2.** The collection and analysis of monitoring data serve to identify vulnerabilities in the system. This is the basis for organizational improvement, risk reduction, and damage control.

*Question 4. How can you prevent and manage Clostridioides difficile infection (CDI)?*

**Statement 4.1.** Key points forCDI prevention are:

- Antimicrobial stewardship.
- Contact precautions.
- Hand washing (soap, not alcohol).
- Avoid unnecessary gastric acid suppressants.

**Statement 4.2.** Key points for CDI treatment are:

- Stop unnecessary antibiotics.
- Metronidazole (mild episodes).
- Oral/intracolonic vancomycin.
- Oral fidaxomicin.
- IV bezlotoxumab (recurrent episodes).
- Fecal microbiota transplantation.

*Question 5. How can you prevent central-venous catheter-related infections?*

**Statement 5.1.** The most effective means to reduce to the minimum possible central-venous catheter-related infections are represented by a «bundles» management, based on the guidelines, implemented with training and motivational meetings aimed at increasing compliance of healthcare workers (better if organized in a dedicated team) and applied by checklist.

*Question 6. The clinical microbiology laboratory: which is its role in the control of infections with multidrug-resistant bacteria?*

**Statement 6.1.** The implementation of microbiological diagnostic activities improves the diagnostic capacity towards infections caused by multidrug-resistant organisms (MDROs).

**Statement 6.2.** The three main challenges of a modern microbiology laboratory are: to maintain high-quality services, to consolidate laboratory medical care into large hospital systems and, as a consequence of consolidation to reach a full automation possibly in all the analytical steps of the diagnosis.

*Question 7. How can you manage the patient with infection/colonization of multidrug-resistant organisms (MDROs)?*

**Statement 7.1.** The application of contact isolation precautions is always recommended for patients known or highly suspected for MDROs.

*Question 8. Antimicrobial stewardship. Is a multidisciplinary approach necessary?*

**Statement 8.1.** The three basic requirements of an Antimicrobial Stewardship program are:

- The existence of a multidisciplinary antimicrobial stewardship team.
- A microbiological report on a fixed basis on the bacterial resistance of the hospital at least annually, if possible stratified by departments or at least for some key departments (e.g. Intensive Care Units).
- A report on fixed consumption of antibiotics in the hospital.

**Statement 8.2.** A multidisciplinary antimicrobial stewardship team should be coordinated by an infectious disease specialist, or by another specialist with documented infectious skills.

*Question 9. Why and how do you have to monitor antibiotics consumption in the hospitals?*

**Statement 9.1.** It is important to monitor antibiotics consumption. The data of the consumption data of antibiotics should be expressed in specific reports in defined daily doses.

*Question 10. What is the role for the nurse in preventing HAIs?*

**Statement 10.1.** The nurse is an integral part of the multidisciplinary team for the prevention of infections across the surgical pathway.

**Statement 10.2.** It is important to implement educational and training interventions concerning the prevention of SSIs by following a modality appropriate to the level of education of the patient/caregiver.

*Question 11. Which are the principles of antibiotic prophylaxis?*

**Statement 11.1.** Prolonging antibiotic prophylaxis after surgery is generally not associated with better clinical results.

**Statement 11.2.** There is no universally recognized intervention for improving the appropriateness of antibiotic prophylaxis in surgery. These interventions must be tailored to the type of surgeon and team to which they are addressed.

*Question 12. Which are the principles of antibiotic therapy?*

**Statement 12.1.** It is important to know the local epidemiological context to define therapeutic protocols / guidelines for surgical infections treatment.

**Statement 12.2.** It is important to frame clinical conditions, in particular to differentiate between critical and non-critical patients.

**Statement 12.3.** It is important to pursue as much as possible targeted therapy or in any case a de-escalation in order to preserve some molecules: e.g.: carbapenems.

**Statement 12.4.** It is important to assess properly the duration of therapy based on source control.

**Statement 12.5.** In the setting of uncomplicated intra-abdominal infections including uncomplicated acute cholecystitis and acute appendicitis post-operative antimicrobial therapy is not necessary.

**Statement 12.6.** In patients with complicated intra-abdominal infections, when patients are not severely ill and when source control is complete, a short course (3-5 days) of post-operative therapy is suggested.

**Statement 12.7.** In patients with ongoing or persistent intra-abdominal infections, the decision to continue, revise, or stop antimicrobial therapy should be made on the basis of clinician judgment and laboratory information.

*Question 13. How can you manage invasive candidiasis in surgical patients?*

**Statement 13.1.** It is important the knowledge of the risk of developing invasive candidiasis, improve microbiological diagnostics and optimize treatment.

*Question 14. Which are the principles of antibiotic therapy in critically ill patients?*

**Statement 14.1.** In critically ill patients, antibiotic therapy should be prescribed using a severity and risk stratification driven approach.

**Statement 14.2.** It is important to support the need for better identification of patients at risk of MDROs infection, more accurate diagnostic tools enabling a rule-in/rule-out approach for bacterial sepsis, the use of adequate dosing and administration schemes to ensure the attainment of pharmacokinetics/pharmacodynamics targets, concomitant source control when appropriate, and a systematic reappraisal of initial therapy in an attempt to minimize collateral damage on commensal ecosystems through de-escalation and treatment-shortening whenever conceivable.

*Question 15. Who is the surgeon champion?*

**Statement 15.1.** To be a champion in preventing and managing infections in surgery means to create a culture of collaboration in which infection prevention, antimicrobial stewardship and correctsurgical approach are of high importance.

*Question 16. Which are the principles of source control?*

**Statement 16.1.** The timing and adequacy of source control are important in the management of surgical infections; late and/or incomplete procedures may have severely adverse consequences on outcome especially in critically ill patients.

*Question 17. What is the role of the biomarkers in surgery?*

**Statement 17.1.** C-reactive protein (CRP) and procalcitonin (PCT) can help clinicians to diagnose surgical infections.

**Statement 17.2.** PCT can help clinicians in early discontinuation of antibiotics in critically ill patients and in patients undergoing intervention for acute peritonitis.

*Question 18. Who are the patients at high risk for surgical site infections?*

**Statement 18.1.** A number of risk factors are known to increase the incidence of SSIs, they can be effective at three different levels: patient, operative (surgical procedure-related) and institutional level (hospital related).

**Statement 18.2.** Although multiple strategies exist for identifying surgical patients at high risk for SSIs, no one strategy is superior for all patients and further efforts are necessary to determine if risk stratification in combination with risk modification can reduce SSIs in this patients’ population.

*Question 19. How can you care post-operative wounds to prevent surgical site infections?*

**Statement 19.1.** Advanced dressing of any type should not be used for primarily closed surgical wounds for the purpose of preventing SSI.

**Statement 19.2.** The surgical wound dressing can be removed for a minimum of 48 hours after surgery unless leakage occurs. There is no evidence that extending medication time implies a reduction in SSIs.

*Question 20. How can we engage surgeons in appropriate infection prevention and management?*

**Statement 20.1.** Active education techniques, such as academic detailing, consensus building sessions and educational workshops, should be implemented in each hospital worldwide according to its own resources.

**Statement 20.2.** Surgeons with satisfactory knowledge in surgical infections should be involved in the infection control team and recognized as “champions” by the hospital’s administration.

## Abbreviations

AMR: Antimicrobial resistance
ASP: Antimicrobial stewardship program
CAUTI: Catheter-associated urinary tract infection
CDC: Centers for Disease Control and Prevention
CDI: *Clostridioides difficile* infection
CLABSI: Central line-associated bloodstream infection
CML: Clinical microbiology laboratory
CRBSI: Central venous catheter-related bloodstream infection
CRP: C-reactive protein
CS: Candida score
CVC: Central venous catheter
DDD: Defined daily dose
ECDC: European Center for Disease Prevention and Control
FMT: Fecal microbiota transplantation
HAI: Healthcare-associated infection
HCW: Healthcare worker
IAT: Inappropriate antibiotic therapy
IC: Invasive candidiasis
ICU: Intensive care unit
IPC: Infection prevention and control
MDRO: Multidrug-resistant organism
PAP: Perioperative antibiotic prophylaxis
PCT: Procalcitonin
PPI: Proton pump inhibitor
SSI: Surgical site infection
WHO: World Health Organization
